# The Characterization of Serum-Free Media on Human Mesenchymal Stem Cell Fibrochondrogenesis

**DOI:** 10.3390/bioengineering12050546

**Published:** 2025-05-19

**Authors:** Ka Yu Carissa Kwan, Ke Li, Yu Yang Wang, Wai Yi Tse, Chung Yan Tong, Xu Zhang, Dan Michelle Wang, Dai Fei Elmer Ker

**Affiliations:** 1 School of Biomedical Sciences, The Chinese University of Hong Kong, Shatin, Hong Kong SAR, China; carissakwan@link.cuhk.edu.hk (K.Y.C.K.); keli@cuhk.edu.hk (K.L.); 1155152082@link.cuhk.edu.hk (Y.Y.W.); 1155143827@link.cuhk.edu.hk (W.Y.T.); athena.tong@link.cuhk.edu.hk (C.Y.T.); xuzhang@cuhk.edu.hk (X.Z.); wangmd@cuhk.edu.hk (D.M.W.); 2 Institute for Tissue Engineering and Regenerative Medicine, The Chinese University of Hong Kong, Shatin, Hong Kong SAR, China; 3 Center for Neuromusculoskeletal Restorative Medicine, Hong Kong Science Park, Shatin, Hong Kong SAR, China; 4 Ministry of Education Key Laboratory for Regenerative Medicine, The Chinese University of Hong Kong, Shatin, Hong Kong SAR, China; 5 Department of Orthopaedics and Traumatology, Faculty of Medicine, The Chinese University of Hong Kong, Shatin, Hong Kong SAR, China; 6 Department of Biomedical Engineering, Faculty of Engineering, The Hong Kong Polytechnic University, Hung Hom, Hong Kong SAR, China

**Keywords:** fibrocartilage tissue engineering, human mesenchymal stem cells, serum-free medium, growth factors, glucose, dexamethasone

## Abstract

Developing fibrochondrogenic serum-free media is important for regenerating diseased and injured fibrocartilage but no defined protocols exist. Towards this goal, we characterized the effect of four candidate fibrochondrogenic serum-free media containing transforming growth factor beta-3 (TGF-β3), insulin-like growth factor-1 (IGF-1), and fibroblast growth factor-2 (FGF-2) with high/low glucose and with/without dexamethasone on human mesenchymal stem cells (hMSCs) via proliferation and differentiation assays. In Ki67 proliferation assays, serum-free media containing low glucose and dexamethasone exhibited the highest growth. In gene expression assays, serum-free media containing low glucose and commercially available chondrogenic media (COM) induced high fibrochondrogenic transcription factor expression (scleraxis/*SCX* and SRY-Box Transcription Factor 9/*SOX9*) and extracellular matrix (ECM) protein levels (aggrecan/*ACAN,* collagen type I/*COL1A1*, and collagen type II/*COL2A1*), respectively. In immunofluorescence staining, serum-free media containing high glucose and COM induced high fibrochondrogenic transcription factor (SCX and SOX9) and ECM protein (COL1A1, COL2A1, and collagen type X/COL10A1) levels, respectively. In cytochemical staining, COM and serum-free media containing dexamethasone showed a high collagen content whereas serum-free media containing high glucose and dexamethasone exhibited high glycosaminoglycan (GAG) levels. Altogether, defined serum-free media containing high glucose exhibited the highest fibrochondrogenic potential. In summary, this work studied conditions conducive for fibrochondrogenesis, which may be further optimized for potential applications in fibrocartilage tissue engineering.

## 1. Introduction

Fibrocartilage regeneration is vital to the repair of injured or diseased connective tissues. As a mechanically tough and fibrous transitional tissue, fibrocartilage is commonly found at bone–tendon/ligament junctions (enthesis), intervertebral discs, and menisci. To efficiently transmit muscle contractile force to bone or resist compressive loads, fibrocartilage is dependent upon the extracellular matrix (ECM)-secreting actions of chondrocytes, producing a dense network of organized collagen fibers and proteoglycans [1,2,3]. Distinct from its cartilage counterparts, fibrocartilage is primarily composed of collagen types I and II whereas hyaline (articular) cartilage principally comprises collagen type II (more than 90%) [3,4,5,6,7]. As a result of its unique molecular composition, fibrocartilage can undergo anisotropic fiber arrangement and increase pyridinoline crosslinking, exhibiting a greater tensile strength (10 MPa) than that of hyaline cartilage (4 MPa) [3,4,5,6,7,8]. Yet, acute tears or chronic degenerative disorders at the spine and joints may lead to a poor prognosis, as invasive surgeries fail to address the high rates of wound re-tear [9]. In addition to fibrovascular scars formed during intrinsic inflammation, the avascular and hypocellular nature of fibrocartilage pose significant challenges for complete regeneration. Indeed, the socioeconomic burden of fibrocartilage injury is significant. For instance, medical costs for meniscus injuries stand at US$4 billion annually [10] while the total cost for degenerative intervertebral disc pain is estimated to be between US$100–200 billion each year [11]. A major barrier in fibrocartilage regeneration stems from our inability to generate sufficient fibrochondrocytes for cell-based therapy. Within this context, it is crucial to develop fibrochondrogenic media absent of ill-defined components such as serum. Such serum-free media formulations are advantageous due to a reduced batch-to-batch variability and lower risk for disease transmission, which facilitate the production of clinical-grade cells for therapeutic use [12]. Therefore, developing well-defined fibrochondrogenic media is essential for cell-based fibrocartilage regeneration.

To satisfy the practical considerations for fibrocartilage tissue engineering, it is crucial to reflect on the source of stem/progenitor cells and the choice of biological cues for inducing fibrochondrogenesis. Human mesenchymal stem cells (hMSCs) are promising cell sources for extensive applications in clinical trials [13] owing to their multipotency, self-renewal ability, ease of extraction from multiple tissue sources [14,15], allogenic immunosuppressive effects, and well-established safety profile [16]. There have been numerous studies on the development of serum-free chondrogenic media [17,18,19,20,21,22,23]. Such investigations together with other serum-based studies have identified crucial mediators of chondrogenesis, including members of the transforming growth factor (TGF) superfamily (TGF-β1, TGF-β3, and bone morphogenetic proteins/BMPs), insulin-like growth factor-1 (IGF-1), fibroblast growth factor-2 (FGF-2), dexamethasone, and glucose [3,17,18,19,20,21,22,23,24,25,26]. However, the majority of such studies have focused on hyaline chondrogenesis [17,18,19,20,21] and what few studies that have focused on fibrochondrogenesis have employed embryonic stem cells [22,23], which are unsuitable for clinical translation due to ethical and safety concerns such as potential teratoma formation. Also, dexamethasone and high glucose have been shown to enhance chondrogenesis in vitro [27,28] but there have been negative reports in vivo relating either to their use [29] or as a result of association with diabetes which causes hyperglycemia [30]. As such, there is still a need to assess the effect of candidate fibrochondrogenic factors to develop MSC-based, serum-free fibrochondrogenic media. Therefore, a comparative study that examines the effects of glucose level and dexamethasone presence is vital for hMSC-based cell therapy for fibrocartilage regeneration.

**Figure 1:**
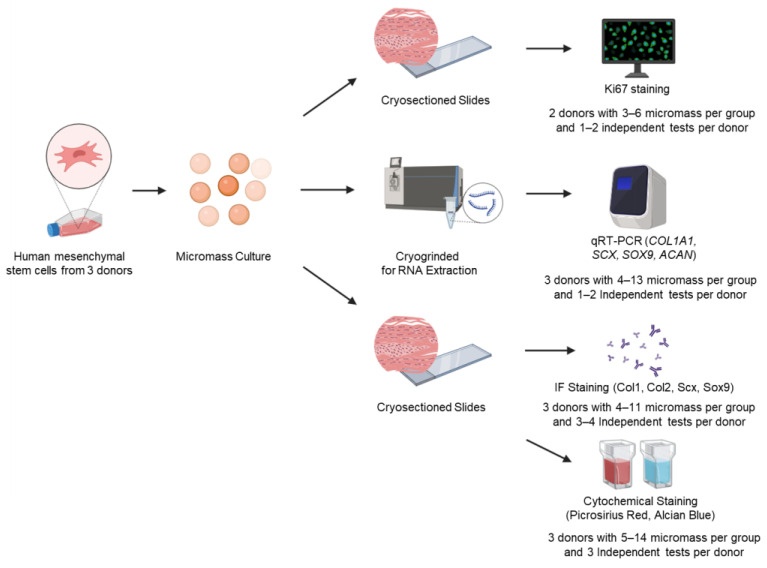
Experimental design to assess hMSC fibrochondrogenic differentiation by serum-free media cocktails. Bone marrow-derived, human mesenchymal stem cells (hMSCs) were used to form high-cell-density micromass pellets and were cultured in various serum-free fibrochondrogenic differentiation media for 7, 14, and 21 days, respectively. Micromass pellets were either cryosectioned or cryogrinded for staining or RNA extraction. Several different approaches were used to investigate hMSC proliferation (Ki67) and fibrochondrogenic differentiation at the gene (qRT-PCR) as well as protein (immunofluorescence and cytochemical staining) levels.

To develop a serum-free fibrochondrogenic media, we examined the effects of a growth factor cocktail containing TGF-β3, IGF-1, and FGF-2 biological factors that have been previously established to be vital for both chondrogenesis [17,19,24,31,32] and tenogenesis [33,34,35] with high/low glucose concentration and the presence/absence of dexamethasone, on hMSCs proliferation and fibrochondrogenesis in vitro. We hypothesized that serum-free media containing TGF-β3, IGF-1, and FGF-2 together with high glucose and dexamethasone would promote hMSC fibrochondrogenesis. Specifically, hMSC proliferation was assessed via Ki67 immunofluorescence staining whereas the gene and protein expression of fibrochondrogenic markers including classical fibrochondrogenic transcription factors SCX and SOX9, and entheseal ECM components COL1A1, COL2A1, and COLX/COL10A1 [36,37,38], were assessed via quantitative real-time polymerase chain reaction (qRT-PCR) and immunofluorescence staining, respectively, with the total collagen and glycosaminoglycan (GAG) deposition quantified via cytochemical staining (Figure 1). In these studies, low glucose media was used as a comparison reference to other fibrochondrogenic media as diabetes (i.e., high glucose levels) has been cited as an important risk factor that impedes successful rotator cuff repair [39] and high glucose media has been used to model this phenomenon in vitro [40].

## 2. Materials and Methods

### 2.1. Cell Culture

Bone marrow-derived hMSCs (up to passage 10) were used in fibrochondrogenic differentiation studies. hMSCs were purchased from Lonza (Cat: PT-2501, Donor: #31286, #34864, #36015, Lot: 0000603525, 0000684888, 18TL169252, Basel, Switzerland) and cultured in growth media (GM; Mesenchymal Stem Cell Growth Media Bullet Kit, Cat: PT-3001, Lonza) as per the manufacturer’s instructions. Cells were passaged using 0.05% trypsin-EDTA (Gibco, Cat: 25300062, Thermo Fisher Scientific Inc., Pittsburgh, PA, USA) when the cell density reached 80% confluency, and they were maintained in a humidified environment at 37 °C under 5% CO_2_ [3].

### 2.2. Fibrochondrogenic Differentiation

To induce fibrochondrogenic differentiation, hMSCs were seeded into individual wells of tissue culture polystyrene grade 48-well plates at a density of 2.5 × 10^5^ cells per 5 µL volume (in GM) and incubated at 37 °C for 1.5 h to form micromass pellet cultures. Thereafter, various culture media were added, and cells were grown for 7, 14, and 21 days, respectively, with a medium change every 3 days. The media included the following groups—(i) commercial serum-free chondrogenic media (COM) as the positive control (Gibco, Cat: A1007101), (ii) low glucose (1 g/L; LG) serum-free fibrochondrogenic differentiation media, (iii) low glucose and dexamethasone (LG + D) serum-free fibrochondrogenic differentiation media, (iv) high glucose (4.5 g/L; HG) serum-free fibrochondrogenic differentiation media, and (v) high glucose and dexamethasone (HG + D) serum-free fibrochondrogenic differentiation media. With the exception of COM, all other serum-free fibrochondrogenic differentiation media included Dulbecco’s Modified Eagle’s Media (DMEM) (high glucose: Gibco, Cat: 11995-065 or low glucose: Gibco, Cat: 12320-032) supplemented with 1% Insulin-Transferrin-Selenium-Ethanolamine (ITS-X) (Gibco, Cat: 51500-056), 1% Penicillin–Streptomycin (10,000 U/mL) solution (Thermo Fisher, Cat: 15140122), 50 μg/mL L-ascorbic acid (Sigma-Aldrich, St. Louis, MO, USA, Cat: A5960-100G), 10 ng/mL TGF-β3 (ProspecBio, Ness-Ziona, Israel, Cat: Cyt 368), 100 ng/mL IGF-1 (ProspecBio, Cat: Cyt216), and 50 ng/mL FGF-2 (ProspecBio, Cat: Cyt 218) with or without 100 nM dexamethasone (Sigma-Aldrich, Cat: D4902) [3].

### 2.3. RNA Extraction and Quantitative Real-Time Polymerase Chain Reaction (qRT-PCR)

RNA extraction was performed using mechanical homogenization [3,17,41], followed by kit-based purification while qRT-PCR was conducted via TaqMan-based nucleic acid amplification as previously described [3,35,42].

For RNA extraction, micromass culture samples were briefly washed in phosphate buffered saline (PBS) (Gibco, Cat: 18912014) twice. Samples were then mechanically homogenized in RLT Plus buffer (supplied by Qiagen RNeasy Mini kit, Hilden, Germany, Cat: 74106) using a Model 6775 Freezer/Mill^®^ Cryogenic Grinder (SPEX SamplePrep, Metuchen, NJ, USA). Mechanical homogenization was performed using two two-minute cycles at maximum frequency with a two-minute cooling in between each cycle to achieve optimal homogenization. Thereafter, the resulting solution was topped up to 350 µL RLT Plus buffer and subsequent steps were performed using Qiagen RNeasy Mini kit in accordance with the manufacturer’s instructions. The total RNA was measured using NanoDrop^®^ ND-2000 (Nanodrop Technologies, Wilmington, DE, USA).

For qRT-PCR, reverse transcription was performed using LunaScript RT Supermix (New England Biolabs, Ipswich, MA, USA, Cat: E3010L) followed by TaqMan-based nucleic acid amplification. Each 10 µL volume of the qRT-PCR reaction comprised 2 ng of sample cDNA template, 5 µL of 2X Luna Universal qPCR Master Mix (New England Biolabs, Cat: M3003S), and 0.5 µL of the respective TaqMan Gene Expression Assay (Thermo Fisher). TaqMan Gene Expression Assays included collagen type I alpha 1 chain (COL1A1) (Cat: Hs00164004_m1), collagen type II alpha 1 chain (COL2A1) (Cat: Hs01064869_m1), scleraxis (SCX) (Cat: Hs03054634_g1), Sry-Box transcription factor 9 (SOX9) (Cat: Hs00165814_m1), and aggrecan (ACAN) (Cat: Hs00153936_m1), and was normalized against 18s ribosomal RNA (RNA18S) (Cat: Hs99999901_s1). qRT-PCR reactions were run in triplicate using a 384-well plate on a QuantStudio™ 7 Pro Real-Time PCR System (Applied Biosystems, Waltham, MA, USA). The PCR cycling conditions were as follows: an initial denaturation at 95 °C for 10 min, followed by 40 cycles of primer annealing and extension at 95 °C for 15 s and 60 °C for 1 min, respectively. Relative expression levels for each primer set were expressed as fold changes by the 2−ΔΔCT method.

### 2.4. Cytochemical and Immunofluorescence Staining

Cytochemical [3,35,43] and immunofluorescence [3,35,42,44] staining was performed as previously described with minor modifications. Micromass pellets were collected at 7 days, 14 days, and 21 days post-seeding, separately. Thereafter, the pellets were briefly rinsed with PBS, embedded in Tissue-Tek O.C.T. compound (OCT) (Sakura Finetek, Torrance, CA, USA, Cat: 4583), and snap-frozen via a 30-s immersion in liquid nitrogen. OCT blocks were then cut into 7-μm sections and mounted onto glass slides for staining.

For cytochemical staining, Picrosirius Red and Alcian Blue staining were performed to assess the collagen and GAG content, respectively. Samples were fixed in 4% paraformaldehyde (PFA; Electron Microscopy Sciences, Hatfield, PA, USA, Cat: 15713S) as necessary. Collagen staining was performed by incubating samples in Picrosirius Red dye (Sigma-Aldrich, Cat: 365548) for 1 h followed by two washes with 0.5% acetic acid (VWR Chemicals, Radnor, PA, USA, Cat: 20104.334) in double-distilled water (ddH_2_O). GAG staining was performed by incubating samples in Alcian blue dye (Electron Microscopy Sciences, Hatfield, PA, USA, Cat: 26116-06) for 30 min followed by three H_2_O washes.

For immunofluorescence staining, cell proliferative marker Ki67 and fibrochondrogenic markers Col1a1, Col2a1, Col10a1, Scx, and Sox9 were used. Ki67 was stained on day 7 and 14. Col1a1, Col2a1, Scx, and Sox9 were stained on day 14 and Col10a1 was stained on day 21. For samples that were fixed using PFA and involved nuclear protein staining (Ki67, Scx, and Sox9), an additional permeabilization step was performed via a 10-min incubation in 0.1% Triton X-100 (Sigma-Aldrich, Burlington, MA, USA, Cat: X100). Samples were blocked with 10% donkey serum in PBS (Merck Millipore, Temecula, CA, USA, Cat: S30-100ML) and incubated at 4 °C overnight with primary antibodies, i.e., 1 μg/mL rabbit anti-Ki67 (Abcam, Cambridge, UK, Cat: Ab15580) or 2 μg/mL rabbit anti-collagen type I (Novus Biologicals, Centennial, CO, USA, Cat: NB600-408), 5 μg/mL mouse anti-collagen type II (Invitrogen, Carlsbad, CA, USA, Cat: MA5-12789) and 5 μg/mL mouse anti-Col10 (Abcam, Cat: Ab49945) or 5 μg/mL rabbit anti-ScxA (Abcam, Cambridge, United Kingdom, Cat: Ab58655) and 10 μg/mL mouse anti-Sox9 (Abcam, Cat: Ab76997). The next day, samples were rinsed three times with antibody wash buffer (0.1% bovine serum albumin in PBS; Sigma-Aldrich, Cat: A2153) for 5 min each and incubated with secondary antibodies for 1 h at room temperature—10 μg/mL donkey anti-rabbit 488 (Invitrogen, Carlsbad, CA, USA Cat: A21206) and 10 μg/mL donkey anti-mouse 647 (Invitrogen, Cat: A31571). Thereafter, samples were rinsed five times with antibody wash buffer for 5 min each, and co-stained with Hoechst 33342 for 15 min (Anaspec, Fremont, CA, USA, Cat: AS-83218).

Images were acquired using an Olympus IX-83 inverted fluorescence microscope (Olympus Life Science, Tokyo, Japan) and Nikon Ti2-E inverted fluorescence microscope (Nikon Instruments, Melville, NY, USA) equipped with a fluorescence light source (Lumencor SPECTRA III Light Engine) under brightfield and fluorescence using appropriate fluorescence filters.

### 2.5. The Semi-Quantification of Cytochemical and Immunofluorescence Images

The semi-quantification of cytochemical and immunofluorescence images was performed using Adobe Photoshop 22.0 (Adobe Systems, San Jose, CA, USA) as previously described [42,44,45]. Briefly, cytochemical microscope images were transformed into grayscale using the ‘Select Color Range’ function and pasted as a grayscale image with high pixel values corresponding to positive dye staining (i.e., red for Picrosirius Red and blue for Alcian Blue). No thresholding or normalization was performed for cytochemical microscope images. Subsequently, a region-of-interest was defined for immunofluorescence and cytochemical images using the marquee tool and the average pixel intensity was measured using the ‘Histogram’ function, which is depicted in the Supplementary Information. For immunofluorescence images, an additional normalization step was performed, dividing the fluorescence signal intensity for the protein-of-interest by the amount of DNA (i.e., Hoechst signal).

### 2.6. Statistical Analysis

Statistical analysis was conducted using SPSS Statistics 23 (IBM, Armonk, NY, USA). For each figure legend, values are represented as mean ± standard error of mean (SEM) and sample sizes for each experiment were indicated. The low glucose without dexamethasone (LG) group was considered as a reference and set to 1.0 in all the experiments. The fold change relative to LG was calculated for the other groups. To confirm the assumptions of normal distribution and equal variance, the Shapiro–Wilk test and the Levene’s test were used, respectively. For gene expression, immunofluorescence, and cytochemistry semi-quantification, one-way analysis of variance (ANOVA) was performed with either Tukey’s post hoc test or Games–Howell post hoc test for multiple comparisons. Statistical significance was established at a *p*-value ≤ 0.05.

## 3. Results

### 3.1. The Effect of Fibrochondrogenic Serum-Free Media on hMSC Proliferation Using Immunofluorescence Staining

**Figure 2:**
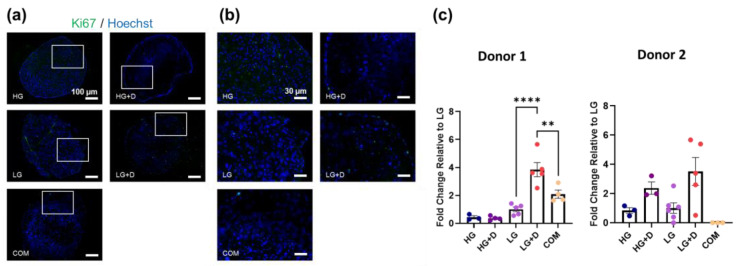
The effect of glucose and dexamethasone on hMSC cell proliferation in serum-free conditions. (**a**) Representative immunofluorescence images of Ki67 and Hoechst 33342-stained hMSCs after 7 days of culture in serum-free fibrochondrogenic media. The area indicated by the white box refers to the magnified inset. (**b**) Magnified inset. (**c**) The semi-quantification of Ki67-positive hMSC numbers. LG was set as a reference and a fold change relative to LG was calculated. HG, high glucose medium; HG + D, high glucose and dexamethasone-containing medium; LG, low glucose medium; LG + D, low glucose and dexamethasone-containing medium; COM, commercial serum-free chondrogenic medium. n = 2 donors, 1–2 replicates with three to six micromass samples for each group in each donor. Error bars indicate SEM. Ki67-positive and Hoechst-stained nuclei are shown in green and blue, respectively. Scale bars as indicated. Statistical significance was established at *p* ≤ 0.05. **, *p* ≤ 0.01; ****, *p* ≤ 0.0001.

hMSC micromass pellets were cultured in four serum-free media combinations containing TGF-β3, IGF-1, and FGF-2, with high/low glucose DMEM (HG and LG) as well as with/without dexamethasone (HG + D and LG + D) and compared to commercial serum-free chondrogenic media (COM). After 7 days of culture, cells were subjected to Ki67 immunofluorescence and Hoechst (DNA) fluorescence staining to visualize proliferating cells and nuclei, respectively (Figure 2a–c).

Ki67 staining (Figure 2a,b) did not show obvious differences among serum-free media groups. Despite some minor variations in donor responses, the semi-quantification analysis of Ki67 staining (Figure 2c) showed a higher staining intensity of Ki67 in the LG + D group (around a 3.8-fold increase in donor 1 and 3.5-fold increase in donor 2, relative to LG), suggesting a higher cell proliferation in LG + D media. Notably, Ki67+ cells were primarily detected at the periphery in dexamethasone-containing serum-free media (HG + D and LG + D) and COM, compared to HG and LG groups. Similar results were observed on 14 days with LG + D media showing a 1.4-fold increase relative to LG media (Supplementary Figure S1). Together, these results showed a limited hMSC proliferation after 7 days of fibrochondrogenic media induction, with proliferating cells primarily located in the superficial zone of hMSC micromass.

### 3.2. The Effect of Serum-Free Media on hMSC Fibrochondrogenic Differentiation Using qRT-PCR of Transcription Factor (TF) and ECM Genes

**Figure 3:**
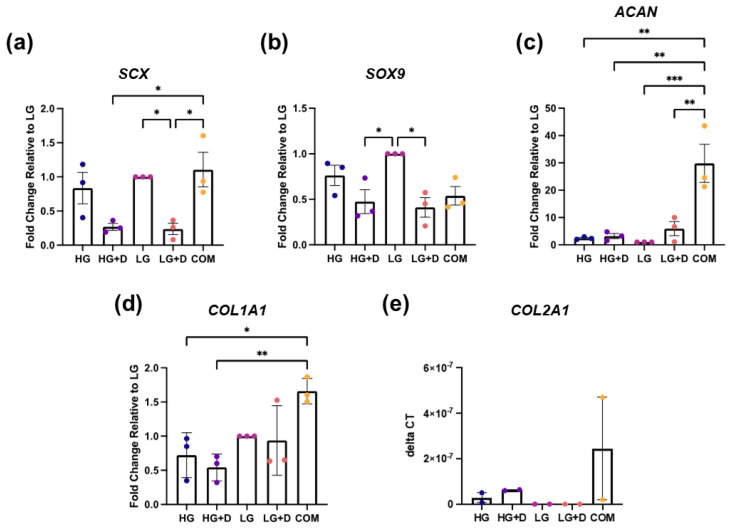
The effect of glucose and dexamethasone on hMSC fibrochondrogenic gene expression. Fibrochondrogenic genes (**a**) SCX, (**b**) SOX9, (**c**) ACAN, (**d**) COL1A1, and (**e**) COL2A1 were assessed with qRT-PCR after 14 days of fibrochondrogenesis. Gene expression was normalized against 18S ribosomal RNA, relative to LG medium. Fold changes were obtained by 2−ΔΔCT method or reported as 2−ΔCT when LG was undetectable. HG, high glucose medium; HG + D, high glucose and dexamethasone-containing medium; LG, low glucose medium; LG + D, low glucose and dexamethasone-containing medium; COM, commercial serum-free chondrogenic medium. n = 3 donors, 1–2 replicates with 4 to 13 micromass samples for each group in each donor. Error bars indicate SEM. Statistical significance was established at *p* ≤ 0.05. *, *p* ≤ 0.05; **, *p* ≤ 0.01; ***, *p* ≤ 0.0001.

The fibrochondrogenic gene expression of hMSC micromass cultures were analyzed using SCX and SOX9 TFs and ACAN, COL1A1 and COL2A1ECM components on day 14 (Figure 3a–e).

For tendon-associated markers, both HG- and LG-containing media showed an increased SCX expression compared to their dexamethasone-containing counterparts (HG + D and LG + D) but a similar COL1A1 expression. Unexpectedly, COM exhibited similar expression levels of SCX as HG and LG groups and a high COL1A1 expression relative to all four experimental fibrochondrogenic media (Figure 3a,d).

For cartilage-associated markers, the LG-containing media group showed a 2.1- and 2.4-fold higher expression of the SOX9 compared to HG + D and LG + D groups, respectively. However, this pattern was not observed for ACAN and COL2A1 expression. Interestingly, COM exhibited the highest ACAN and COL2A1 expression (Figure 3b,c,e, and Supplementary Figure S2).

These results showed that LG-containing media and COM induced the highest fibrochondrogenic TF and ECM gene expression, respectively.

### 3.3. The Effect of Serum-Free Media on hMSC Fibrochondrogenic Differentiation Using Immunofluorescence Staining of TF and ECM Proteins

**Figure 4:**
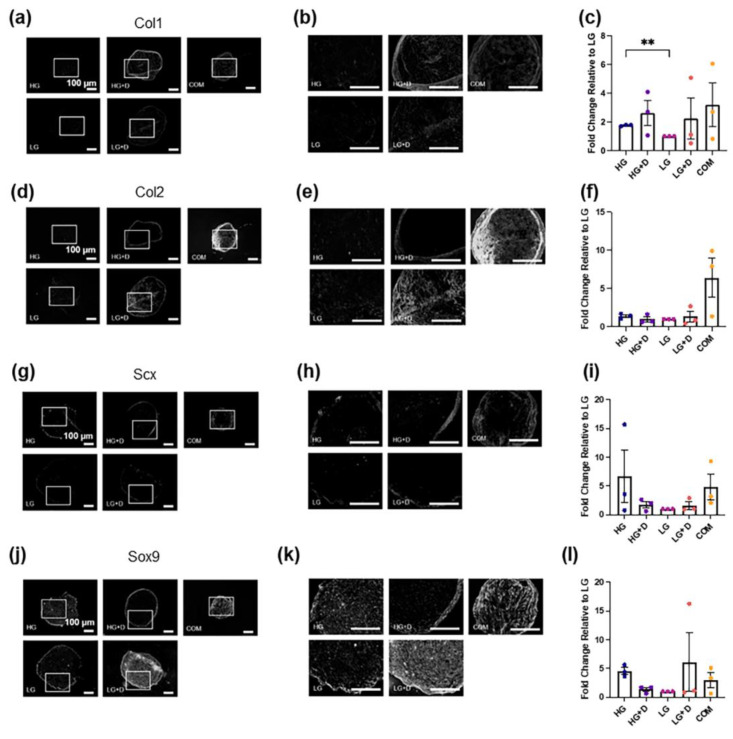
The effect of glucose and dexamethasone on hMSC fibrochondrogenic ECM protein and transcription factor levels. (**a**) Fibrochondrogenic ECM protein Col1a1 was assessed with immunofluorescence staining after 14 days of fibrochondrogenesis. The area indicated by the white box refers to the magnified inset. (**b**) Magnified inset. (**c**) The semi-quantification of Col1a1-positive signals. (**d**) Fibrochondrogenic ECM protein Col2a1 was assessed with immunofluorescence staining after 14 days of fibrochondrogenesis. The area indicated by the white box refers to the magnified inset. (**e**) Magnified inset. (**f**) The semi-quantification of Col2a1-positive signals. The fold changes of immunofluorescence intensity were relative to LG medium. (**g**) Fibrochondrogenic transcription factor Scx was assessed with immunofluorescence staining after 14 days of fibrochondrogenesis. The area indicated by the white box refers to the magnified inset. (**h**) Magnified inset. (**i**) The semi-quantification of Scx-positive signals. (**j**) Fibrochondrogenic transcription factor Sox9 was assessed with immunofluorescence staining after 14 days of fibrochondrogenesis. (**k**) The area indicated by the white box refers to the magnified inset. (**l**) The semi-quantification of Sox9-positive signals. The fold changes of immunofluorescence intensity were relative to LG medium. HG, high glucose medium; HG + D, high glucose and dexamethasone-containing medium; LG, low glucose medium; LG + D, low glucose and dexamethasone-containing medium; COM, commercial serum-free chondrogenic medium. n = 3 donors, 3–4 technical replicates with 4 to 11 micromass samples for each group in each donor. Error bars indicate SEM. Statistical significance was established at *p* ≤ 0.05. **, *p* ≤ 0.01. Scale bar–100 µm.

To complement gene expression studies, Col1a1, Col2a1, and Col10a1 (Figure 4a–f and Supplementary Figure S3 and S4) as well as Scx and Sox9 (Figure 4g–l) protein levels were assessed using immunofluorescence staining and were semi-quantified.

For ECM proteins, the HG and HG + D group enhanced the Col1a1 levels by about 2- and 2.6-fold, respectively, compared to the LG group (Figure 4a–c), whereas little difference was observed for Col2a1 levels among the four serum-free media (Figure 4d–f). Notably, dexamethasone-containing media groups (HG + D and LG + D) showed stronger Col1a1 and Col2a1 signals at the periphery of micromass cryosections, respectively (Figure 4b,e). Interestingly, COM resulted in the highest Col1a1 and Col2a1 deposition (Figure 4c,f) although spatial heterogeneity was noted (Supplementary Figure S3). For example, HG media showed the highest Col1a1 levels (about a 3.5-fold increase compared to LG) and comparable levels of Col2a1 compared to COM in the central regions of the micromass (Supplementary Figure S3). Also, COM showed the highest Col2a1 deposition in both central and peripheral regions of the micromass (Supplementary Figure S3). Additionally, the HG + D group showed the highest Col10a1 levels among the four serum-free media, and was found to be around 1.86-fold higher than COM (Supplementary Figure S4).

For TF proteins, Scx signals were not highly expressed across among the four serum-free media and COM groups (Figure 4g–i). High glucose-containing media (HG and HG + D) and COM groups showed an enhanced Scx protein synthesis relative to low glucose-containing media groups (LG and LG + D) (Figure 4g–i). Sox9 signals were higher in HG and LG + D groups relative to the HG + D, LG, and COM groups (Figure 4j–l).

These results showed that HG media and COM induced the highest fibrochondrogenic TF and ECM protein levels, respectively.

### 3.4. The Effect of Serum-Free Media on hMSC Fibrochondrogenic Differentiation Using the Total Collagen and GAG Content

**Figure 5:**
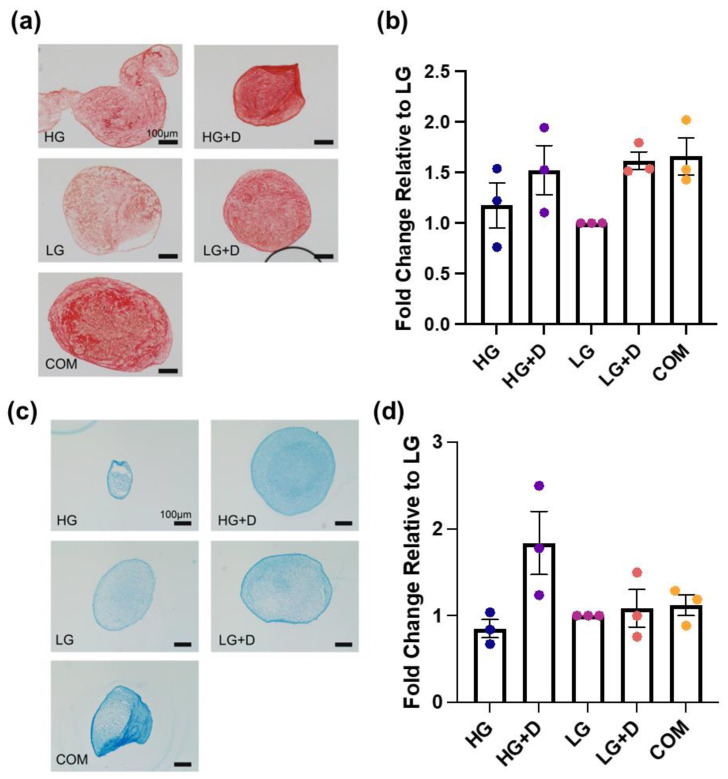
The total collagen and total GAG levels visualized by Picrosirius Red and Alcian Blue staining with brightfield imaging. (**a**) The total collagen levels were assessed with Picrosirius Red staining after 14 days of fibrochondrogenesis. (**b**) The semi-quantification of Picrosirius Red-positive signals. The fold changes of Picrosirius Red staining intensity were relative to LG medium, after 14 days of fibrochondrogenesis. (**c**) The total GAG levels were assessed with Alcian Blue staining after 14 days of fibrochondrogenesis. (**d**) The semi-quantification of Alcian Blue-positive signals. The fold changes of Alcian Blue staining intensity were relative to LG medium, after 14 days of fibrochondrogenesis. HG, high glucose medium; HG + D, high glucose and dexamethasone-containing medium; LG, low glucose medium; LG + D, low glucose and dexamethasone-containing medium; COM, commercial serum-free chondrogenic medium. n = 3 donors, three independent replicates with 5 to 14 micromass samples for each group of each donor. Error bars indicate SEM. Scale bar—100 µm.

To complement gene and protein expression studies, the total collagen (Figure 5a,b) and GAG (Figure 5c,d) content were assessed using Picrosirius Red and Alcian Blue staining, respectively (Supplementary Figure S5).

The total collagen assessment indicated that dexamethasone-containing media (HG + D and LG + D) and COM had a comparable effect in promoting the total collagen deposition, which was higher than non-dexamethasone-containing media (HG and LG) (Figure 5a,b). Visually, the intense and uniform positive staining was apparent throughout the whole pellet and formed a more circular and structured collagen network extending towards the center of the micromass pellet (Figure 5a). Notably, dexamethasone-containing media (HG + D and LG + D) and COM showed about a 1.5-fold increase in collagen compared to LG (Figure 5a).

The GAG assessment indicated that HG + D induced the highest levels of GAG production, with about a 1.8-fold increase relative to LG (Figure 5c,d). Meanwhile, dexamethasone-containing media (HG + D and LG + D) and COM all showed a relatively lower and comparable increased GAG content. Interestingly, a higher GAG expression was noted at the periphery of the micromass pellets (Figure 5c).

These results showed that dexamethasone-containing media and COM induced the highest collagen content while HG + D induced the highest GAG levels.

### 3.5. The Identification of Serum-Free Media with the Greatest Fibrochondrogenic Potential

Owing to different assessment outcomes from multiple assays and inconsistencies arising from donor-to-donor variability, an unweighted scoring system was used to evaluate the best fibrochondrogenic inductive media. In order to reflect the overall effects of each serum-free media formulation on fibrochondrogenesis, scores from the highest mark 5 to the lowest mark 1 were assigned to each group according to the result for each individual parameter in all the assessments performed. Among our four serum-free media formulations, HG was identified to have the greater fibrochondrogenic potential, whilst COM unexpectedly exhibited the greatest fibrochondrogenic effect (Table 1).

## 4. Discussion

Increasing efforts have been made on elucidating major constituents of serum-free tenogenic and chondrogenic media, including TGF-β3, IGF-1, FGF-2, dexamethasone, ITS-X, and ascorbic acid [17,18,19,20,21,22,23,34,46]. Yet, the ambiguous identity of fibrochondrocytes and their inability to robustly regenerate is worthy of further investigation on fibrochondrogenic cues. In this study, we investigated the effects of a potential fibrochondrogenic growth factor cocktail (TGF-β3, IGF-1, and FGF-2) together with a high/low glucose concentration as well as the presence/absence of dexamethasone on hMSC fibrochondrogenic differentiation in micromass cultures. Using a panel of gene, protein, and cytochemical assessments (Figure 1), the results indicated that serum-free media containing TGF-β3, IGF-1, and FGF-2 with a high glucose concentration and no dexamethasone (HG) was the most optimal formulation out of the four serum-free media but did not perform as well as COM (Table 1). Interestingly, HG serum-free media also induced the highest levels of Scx and Sox9 TFs but lower levels of ECM components than COM at both gene and protein levels (Figure 3 and Figure 4). This suggests that a further optimization of this serum-free growth factor combination (TGF-β3, IGF-1, FGF-2, and dexamethasone) may improve the fibrochondrogenic induction ability. This is particularly important since Scx protein levels were found to be relatively low in all four serum-free media formulations as well as COM (Figure 4). An unweighted scoring system was used, as both proliferation and differentiation are crucial for attaining adequate numbers of specialized fibrochondrocytes typically required for regenerative therapies. Such efforts may include the co-delivery of fibrochondrogenic media and hMSCs with low-cost hydrogels that have tunable physiocochemical attributes [47,48].

Growth factors such as TGF-β3, IGF-1, and FGF-2 play important roles in the induction of fibrochondrogenesis. Prior developmental studies have demonstrated that fibrocartilage tissues such as bone–tendon junctions are formed from progenitor cells that are both Scx- and Sox9-positive [49], indicating that fibrochondrocyte progenitors exhibit a combination of tendon- and cartilage-like phenotypes. TGF-β signaling is crucial for both tenocyte [34,35,50,51] and chondrocyte [52] differentiation, and has been used to induce fibrochondrogenesis on nanofibrous biomaterials [53]. Likewise, FGF signaling [32,33,34,42,44,54] and IGF signaling [31,33,34] also contribute towards tenocyte and chondrocyte identities. For example, the targeted deletion of FGF signaling in mouse tendons negatively impacted enthesis (fibrocartilage) development, as evidenced by disrupted enthesis mineralization [55]. In addition, the delivery of modified pegylated IGF-1 in a rat model of a rotator cuff injury resulted in the re-establishment of bone-to-tendon enthesis structure with improved tensile properties [56]. Therefore, our study utilized TGF-β3, IGF-1, and FGF-2 in our growth factor cocktail.

Glucose is the main energy substrate and precursor for ECM protein and GAG production in chondrocytes [57], and promotes chondrogenic differentiation [58]. However, recent studies have also shown that a high glucose level (4.5 g/L, 25 mM) induces MSCs senescence, apoptosis, and significantly downregulates stemness gene expressions [59,60], which could severely impact the quality of differentiated cells. Clinically, a high glucose environment in diabetic tendinopathy leads to low-grade chronic inflammation, poor tendon healing, and a higher re-tear rate, which is directly relevant to fibrochondrogenic interfacial tissue regeneration [61,62,63]. The human physiological range of glucose levels is approximately 3.9–7.1 mM (70–130 mg/dL), and therefore high (4.5 g/L, 25 mM) and low glucose (1 g/L, 5.5 mM) concentration media used in this study can be associated with blood glucose levels of diabetic and healthy patients, respectively. Indeed, there are studies [57,64] that indicate high glucose alone resulted in inefficient chondrogenesis. Despite this, there are also investigations that showed that high glucose levels (17.5 mM) enhanced aggrecan expression in mouse chondrocytes, which is mediated through the protein kinase Cα (PKCα)- and p38-dependent pathway [65].

Dexamethasone is known to play a significant role in inducing hMSC proliferation [66] and differentiation including chondrogenesis [67,68]. Studies have shown that dexamethasone supported hMSC stemness upon serial passaging including proliferation potential as well as enhanced growth-factor mediated hMSC chondrogenesis and rabbit chondrocyte neocartilage formation in vivo [69,70]. However, the long-term systemic injection of anti-inflammatory corticosteroids like dexamethasone exacerbates tendinitis, resulting in impaired tendon healing and rupture [71,72,73]. Notably, low concentrations of dexamethasone (100 nM) have been found to increase hMSC proliferation and osteoblast differentiation including a reduction in donor-to-donor variations [74]. Yet, some donor-to-donor variation was still observed for proliferation (Figure 2c), which may be further addressed with transient dexamethasone supplementation [74]. Also, 100 nM dexamethasone has also been reported to increase chondrogenic differentiation and may prevent apoptosis under high-density culture conditions. A previous study has shown that hMSCs supplemented with high glucose DMEM and dexamethasone increased Col2a1 and aggrecan production [68], which is in agreement with HG + D (100 nM), exhibiting the highest level of Alcian Blue staining (Figure 5) and comparable high levels of Picrosirius Red staining in HG + D and LG + D (Figure 5). These results are also reflected by the upregulated Col1a1 expressions (Figure 4) and resonate with prior chondrogenic studies [75,76]. Therefore, our studies employed low concentrations (100 nM) of dexamethasone.

Several limitations to our study exist. Although we have identified HG exhibiting a greater fibrochondrogenic potential, COM was surprisingly effective for fibrochondrogenesis and there were few tenogenic/fibroblast signals as evidenced by the low Scx and Col1a1 signals among most of the groups (Figure 4). While this may be partly attributed to MSC donor functional differences [77], additional proliferation and differentiation assessments at early (e.g., 3–5 days) and late (e.g., 21 days) timepoints along with more donors may provide a more comprehensive and robust characterization of our findings. Also, in addition, our serum-free media formulation does not contain albumin, which has often been incorporated as a crucial component in serum-free media [78]. As albumin can interact positively with ligands such as lipids, growth factors to influence cell behavior, the inclusion of recombinant albumin may have the potential to promote cell survival and differentiation. In addition, recent single-cell RNA-seq studies have identified retinoic acid signaling as playing a major role in fibrochondrogenic and tenogenic fate switch [22], and may be further considered as a biological cue in optimizing our serum-free media. Future iterations of the media formulation could optimize growth factor concentrations as well as incorporate molecules such as albumin and retinoic acid to enhance fibrochondrogenesis. Despite these limitations, our serum-free media formulations are defined with a known composition in contrast to COM, which remains proprietary and undisclosed in terms of its formulation. Indeed, this work has potential benefits for the induction of fibrochondrogenesis in hMSCs, a clinically promising source of multipotent stem cells, which will complement tissue engineering strategies that seek to combine stem cells with advanced biomaterials [70,71] as well as previous studies utilizing serum-free media in embryonic and induced pluripotent stem cells [22].

## 5. Conclusions

In conclusion, we evaluated the fibrochondrogenic potential of various serum-free media containing TGF-β3, IGF-1, and FGF-2 with different glucose levels and dexamethasone presences, and found that media containing HG exhibited a superior chondrogenic ability. While these media did not outperform commercial chondrogenic media, their defined composition is valuable for fibrochondrogenesis research and may be optimized further to benefit fibrocartilage tissue engineering.

## Figures and Tables

**Table 1:** A scoring system to evaluate fibrochondrogenic media based on multiple assay outcomes. For scoring, highest = 5, lowest = 1. The same ranks were assigned for similar outcomes. Higher scores denote more optimal serum-free fibrochondrogenic media.

	HG	HG + D	LG	LG + D	COM
**Cell Proliferation**					
Ki67	3	3	3	5	4
**qRT-PCR**					
*COL1A1*	3	3	4	4	5
*COL2A1*	3	4	/	/	5
*SCX*	5	4	5	4	5
*SOX9*	4	3	5	3	3
*ACAN*	4	4	3	4	5
**IF Staining**					
Col1a1	4	4	3	4	5
Col2a1	4	4	4	4	5
Col10a1	4	5	3	4	2
Scx	5	3	3	3	4
Sox9	4	2	2	5	3
**Cytochemical Staining**					
Picrosirius Red	3	4	2	5	5
Alcian Blue	4	5	4	4	4
**Total Score**	50	48	41	49	55

## Data Availability

The data presented in this study are available on reasonable request from the corresponding author.

## Supplementary Materials

The following supporting information can be downloaded at: https://www.mdpi.com/article/10.3390/bioengineering12050546/s1, Figure S1: Effect of glucose and dexamethasone on hMSC cell proliferation in serum-free conditions; Figure S2: Effect of glucose and dexamethasone on hMSC fibrochondrogenic gene COL2A1 expression; Figure S3: Effect of glucose and dexamethasone on hMSC fibrochondrogenic ECM proteins in central and peripheral regions of the micromass; Figure S4: Effect of glucose and dexamethasone on hMSC fibrochondrogenic ECM protein Col10a1 expression; Figure S5: Semi-quantification of cytochemical images using Adobe Photoshop.
