# Supplementary material for: The Characterization of Serum-Free Media on Human Mesenchymal Stem Cell Fibrochondrogenesis
Source: Bioengineering (Basel). 2025 May 19;12(5):546. doi: 10.3390/bioengineering12050546 (PMC12109459; doi:10.3390/bioengineering12050546)
Supplement: Supplementary file 1 [file bioengineering-12-00546-s001.zip › bioengineering-3579535-supplementary.pdf]

Supplementary Information

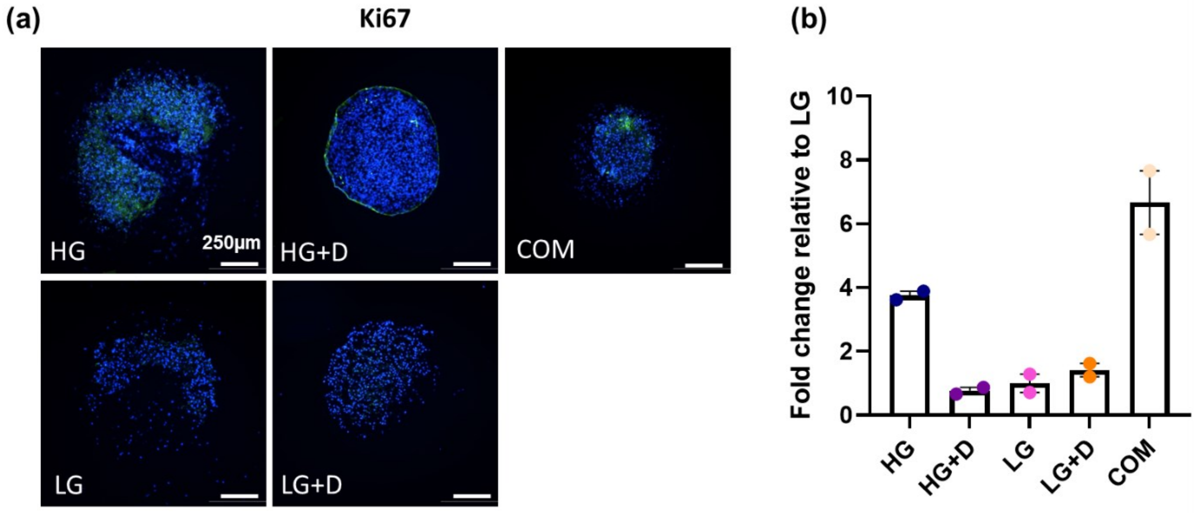

Supplementary Figure S1. Effect of glucose and dexamethasone on hMSC cell proliferation in serum-free conditions. (a) Representative immunofluorescence images of Ki67 and Hoechst 33342-stained hMSCs after 14 days of culture in serum-free fibrochondrogenic media. Ki67-positive and Hoechst-stained nuclei are shown in green and blue respectively. Scale bars as indicated. (b) Semi-quantification of Ki67-positive signals. HG, high glucose medium; HG+D, high glucose and dexamethasone-containing medium; LG, low glucose medium; LG+D, low glucose and dexamethasone-containing medium; COM, commercial serum-free chondrogenic medium. n = 2 micromass per group from 1 donor.

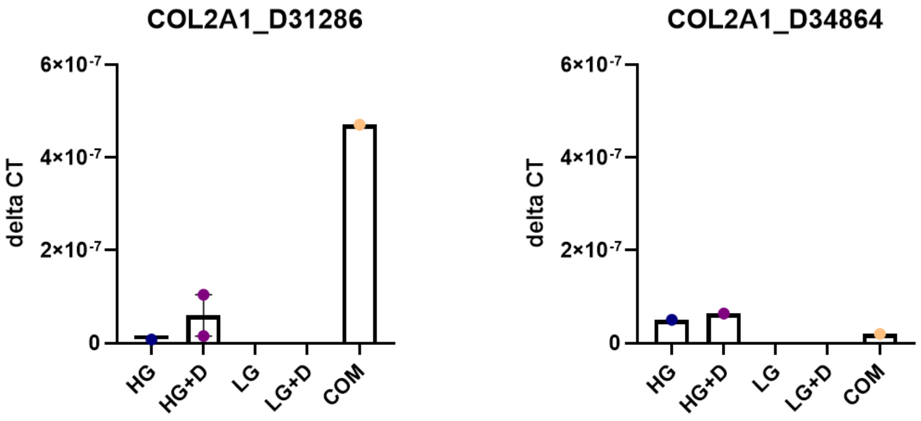

Supplementary Figure S2. Effect of glucose and dexamethasone on hMSC fibrochondrogenic gene COL2A1 expression.

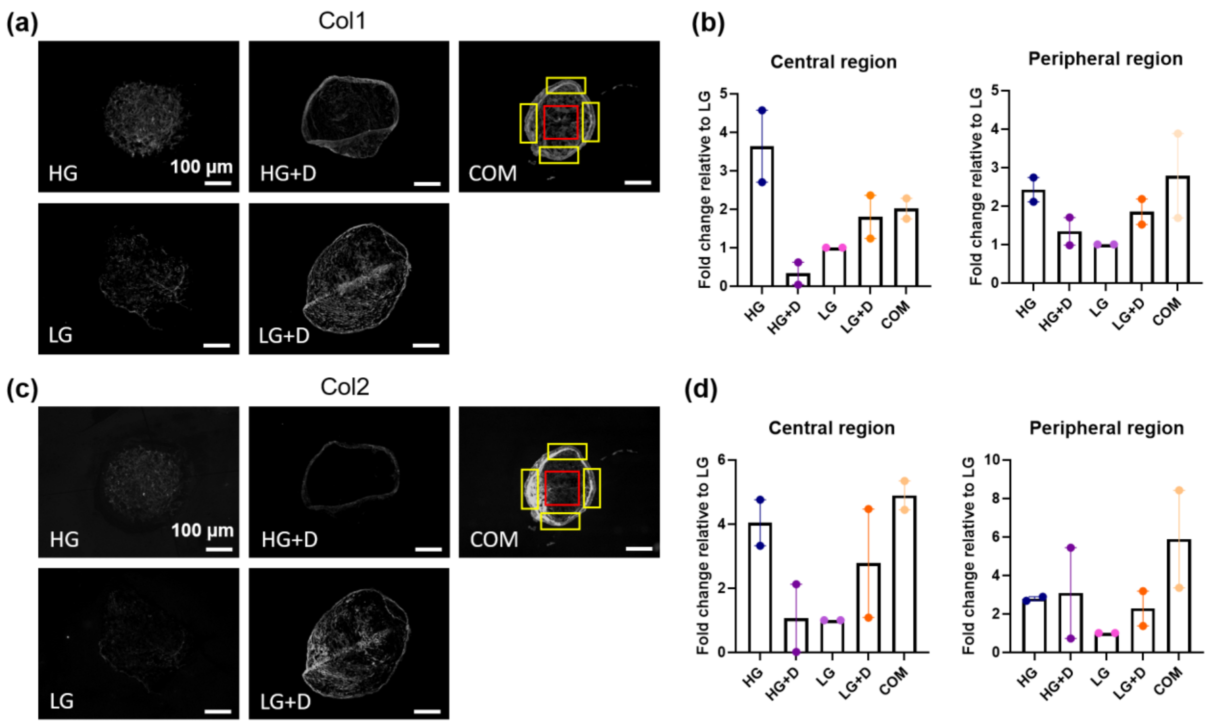

Supplementary Figure S3. Effect of glucose and dexamethasone on hMSC fibrochondrogenic ECM proteins in central and peripheral regions of the micromass. (a) Fibrochondrogenic ECM protein Col1 was assessed with immunofluorescence staining after 14 days of fibrochondrogenesis. Red square represents the central region. Yellow rectangles represent the peripheral regions. (b) Semi-quantification of Col1-positive signals. (c) Fibrochondrogenic ECM protein Col2 was assessed with immunofluorescence staining after 14 days of fibrochondrogenesis. Red square represents the central region. Yellow rectangles represent the peripheral regions. (d) Semi-quantification of Col2-positive signals. Fold changes of immunofluorescence intensity were relative to LG medium. HG, high glucose medium; HG+D, high glucose and dexamethasone-containing medium; LG, low glucose medium; LG+D, low glucose and dexamethasone-containing medium; COM, commercial serum-free chondrogenic medium. n = 2 donors, 1 replicate per donor. Error bars indicate SEM.

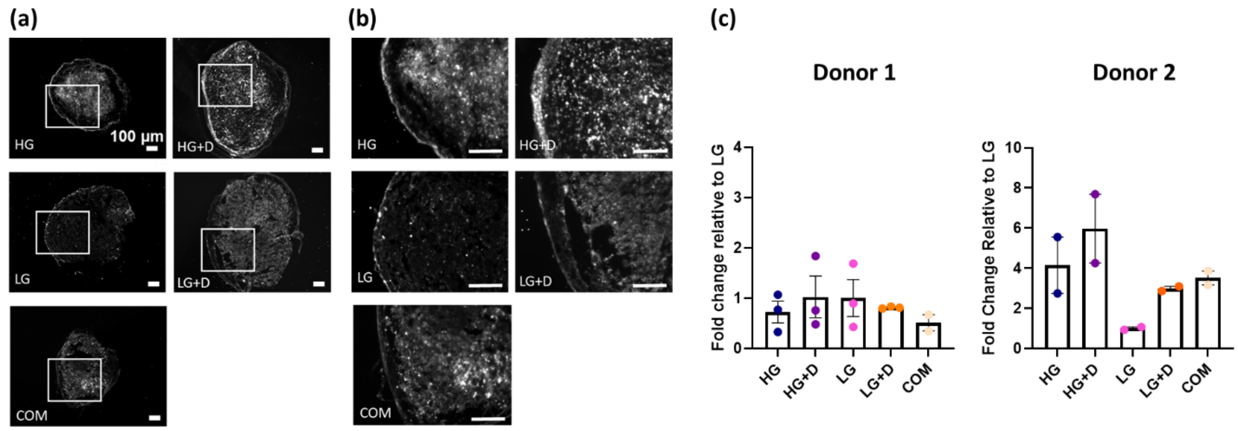

Supplementary Figure S4. Effect of glucose and dexamethasone on hMSC fibrochondrogenic ECM protein Col10 expression. (a) Fibrochondrogenic ECM protein Col10 was assessed with immunofluorescence staining after 21 days of fibrochondrogenesis. The area indicated by the white box refers to the magnified inset. (b) Magnified inset. (c) Semi-quantification of Col10-positive signals from different donors. Fold changes of immunofluorescence intensity were relative to LG medium. HG, high glucose medium; HG+D, high glucose and dexamethasone-containing medium; LG, low glucose medium; LG+D, low glucose and dexamethasone-containing medium; COM, commercial serum-free chondrogenic medium. n = 2 micromass per donor. Error bars indicate SEM.

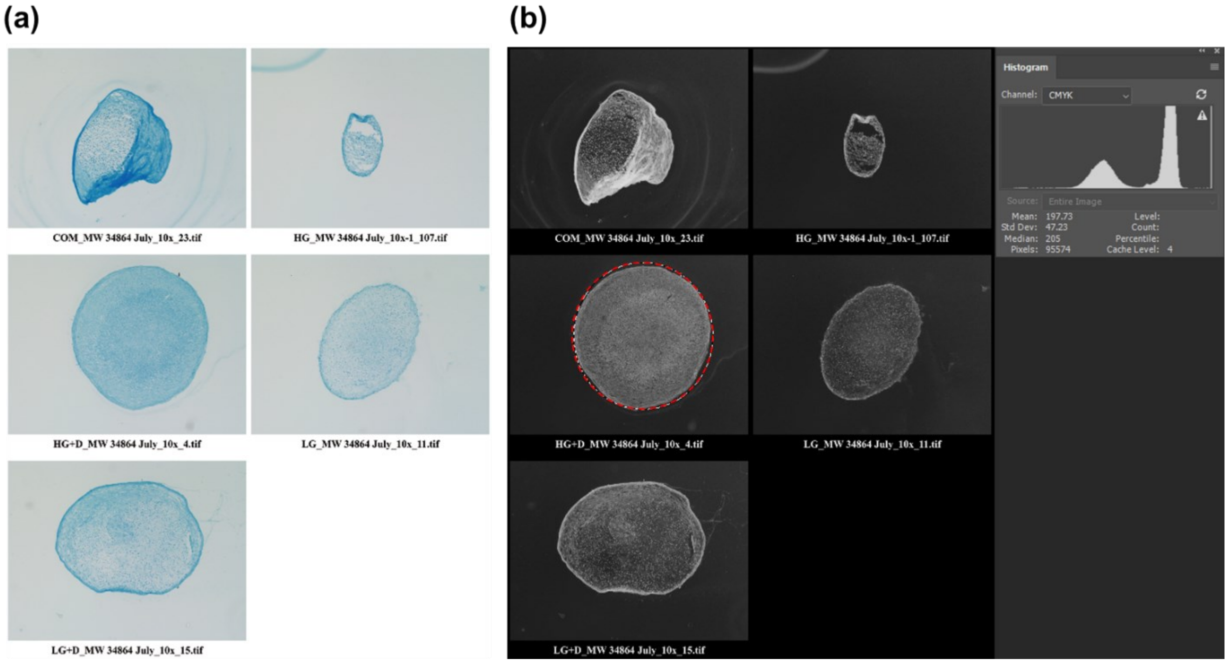

Supplementary Figure S5. Semi-quantification of cytochemical images using Adobe Photoshop. (a) Representative images of Alcian Blue staining after 14 days of fibrochondrogenesis. (b) Converted grayscale images using the "Select Color Range" function corresponding for Alcian Blue staining. Red dot circle represents the selected region-of-interest using the marquee tool and average pixel intensity was measured using the 'Histogram' function.
